# Isoniazid, Pyrazinamide and Rifampicin Content Variation in Split Fixed-Dose Combination Tablets

**DOI:** 10.1371/journal.pone.0102047

**Published:** 2014-07-08

**Authors:** Thomas Pouplin, Pham Nguyen Phuong, Pham Van Toi, Julie Nguyen Pouplin, Jeremy Farrar

**Affiliations:** 1 Mahidol University-Oxford Tropical Medicine Research Programme, Faculty of Tropical Medicine, Mahidol University, Rajthevee, Bangkok, Thailand; 2 Centre for Tropical Medicine, Oxford University Clinical Research Unit Vietnam, Wellcome Trust Major Overseas Programme, Ho Chi Minh City, Vietnam; 3 Centre for Tropical Medicine, Nuffield Department of Medicine, University of Oxford, Oxford, United Kingdom; Concordia University Wisconsin, United States of America

## Abstract

**Setting:**

In most developing countries, paediatric tuberculosis is treated with split tablets leading to potential inaccuracy in the dose delivery and drug exposure. There is no data on the quality of first-line drugs content in split fixed-dose combination tablets.

**Objective:**

To determine Isoniazid, Pyrazinamide and Rifampicin content uniformity in split FDC tablets used in the treatment of childhood tuberculosis.

**Design:**

Drug contents of 15 whole tablets, 30 half tablets and 36 third tablets were analysed by high performance liquid chromatography. The content uniformity was assessed by comparing drug content measured in split portions with their expected amounts and the quality of split portions was assessed applying qualitative specifications for whole tablets.

**Results:**

All whole tablets measurements fell into the USP proxy for the three drugs. But a significant number of half and third portions was found outside the tolerated variation range and the split formulation failed the requirements for content uniformity. To correct for the inaccuracy of splitting the tablets into equal portions, a weight-adjustment strategy was used but this did not improve the findings.

**Conclusion:**

In split tablets the content of the three drugs is non-uniform and exceeded the USP recommendations. There is an absolute need to make child-friendly formulations available for the treatment of childhood tuberculosis.

## Introduction

Tuberculosis (TB) is an increasing global public health challenge which is highly dependent on social and economic factors that significantly affect health care delivery. Tuberculosis in childhood is neglected with the evidence for treatment and clinical care mostly extrapolated from studies in adults [1].

Since the early years of anti-TB drug development, children have been largely excluded from major clinical trials. As a consequence, although some advances have been made, the evidence base on which treatment of childhood TB is determined is weak and the recommendations in childhood TB remain based on extrapolation from observations in adult patients [1]–[3]. The World Health Organisation (WHO) recognised the problem of potential under-dosing in children, especially for isoniazid (INH) and rifampicin (RIF) [4]–[6]. The dose regimen recommendation in children was amended in 2010 and the dose of all the first-line anti-TB drugs increased [7]. The revised regimen for childhood TB recommends the following dosage for the first-line therapy: Isoniazid 10 mg/kg, Rifampicin 15 mg/kg, Pyrazinamide (PYZ) 35 mg/kg and Ethambutol (EMB) 20 mg/kg. In the absence of a pediatric formulation, only the adult FDC formulation, designed for 15 kilos of body-weight, is used instead. When patient's body weight stands below 15 kilos, tablets are split in halves or third and in extremely rare case (i.e. new born) quarters are to be used. This regimen is still based on a body-weight scale, although for the youngest patients, body surface area scale has been proposed to enhance the exposure [5], [8]. However, the new paediatric TB regimen is complicated by the fact that the new drugs ratio is not compatible with the existing FDC. Supplementary single drug tablets have to be added to the treatment, sometimes doubling the pills load with all the consequences on the treatment compliance. In most of the high-burden countries, whilst HIV antiretroviral paediatric formulations have been developed, child-friendly preparations for TB are not available and the paediatric population is treated by splitting fixed-dose combination (FDC) tablets. In tablets, active ingredients are confined and protected by a film-coating process, but the drug may not be uniformly distributed in split portions. Breaking this layer also increases the surface area, affects the stability of drugs and affects the bioavailability. This is especially expected for RIF, the most unstable of the three anti-TB drugs contained in the FDC tablets [9], [10]. In the absence of paediatric liquid formulation, the least expected are FDC tablets aimed for lower body weight band (i.e. 5 kg) in order to avoid the tablet splitting step. Currently, no quality assured version of such products is known to be readily available.

In childhood TB, dividing tablets may lead to dose inaccuracy, weakening the active ingredients and by complicating the delivery may interfere with adherence over the protracted period of therapy. The literature lacks data or guidelines on the evaluation of the content uniformity in split tablets, assuming as a consequence that the weight and drug content among the split fractions used in the treatment are uniforms. International medicine agencies provide equivalent reference guidelines for the evaluation of the dosage uniformity in pharmaceuticals [11], [12]. However, those official pharmacopoeia monographs aim to control only the uniformity of weight and content in intact dosage units (single or FDC tablets), while there is neither recommendation for the assessment of the drug dosage uniformity in tablet fractions, nor evidence that they would be appropriate for the analysis of split portions.

The variability imposed on the dosing by splitting tablets is speculative and this study sought to assess whether the drug content and consequently the drug exposure in TB patients was affected by splitting fixed dose tablets designed for adults but administered to children. We believe this is the first study to investigate the uniformity of INH, Pyrazinamide (PZA) and RIF in split FDC tablets.

## Materials and Methods

### Test formulation and splitting process

The three drugs FDC formulation tested were locally produced film-coated, scored tablets containing 150 mg of RIF, 75 mg of INH and 400 mg of PZA. Each tablet is designed for 15 kg of body-weight and uses a drug ratio compatible with the adult TB regimen. We first assessed different cutting techniques (manual, pill splitter and cutter knife). The cutter knife was used in this study because producing significantly smaller fragments. It was also more convenient to split the tablets into visually equal portions. More than all, this is the technique of choice in pediatric wards in Ho Chi Minh City.

### Reagents and apparatus

All reagents and solvents used were of analytical grade. The reference standards INH (100%), PZA (>99%) and RIF (95.1%) were purchased from Sigma-Aldrich, Singapore. The liquid chromatography system was a Hitachi Lachrom Elite (VWR - Merck, Vietnam) composed by an organizer, an autosampler L-2200, two pumps L-2130, a column Oven L-2350 and a diode array detector (DAD) L-2455. The system was piloted by EZchrom Elite version 3.18 HPLC System Manager Software (Scientific software Inc., San Ramon, USA). The analysis was performed on a 5 µm LichroCart 125×4 mm Purosphere Star RP-8 end-capped column, equipped with a 5 µm guard column Lichrocart 4×4, RP-18e (Merck, Darmstadt, Germany).

### Chromatographic conditions and validation

The mobile phase for INH-PZA consisted in a phosphate buffer 50 mM (pH 4.2) - ACN (99∶1, v/v), and for RIF in a phosphate buffer 50 mM (pH 4.2) - ACN (60∶40, v/v) mixture. Both phases were filtered and then degassed for 30 minutes in a sonic bath. The autosampler temperature was set at 6°C and the injection volume was 10 µL. The chromatography was performed at 35°C in 7 min for INH, PZA (263 nm) and 5 min for RIF (335 nm) at a flow rate of 1 mL/min. Selectivity was assessed in the presence of INH, PZA and RIF in both solutions. None interferences were found from the formulation excipients. Both assays for INH-PZA and RIF were linear (r^2^>0.998) in the range of 70 to 130% of the expected labelled content. The intra- and inter-day precisions were less than 3% and the accuracy remained within 100±4% for the three drugs at three different concentrations (80, 100 and 120% of the expected quantities). A system suitability test was performed prior to any sample analysis with a tolerated variation on area response and retention time of less than 2%.

### Preparation of test solutions

Randomly selected tablets were individually weighted using a calibrated analytical balance (Sartorius AG, Germany). Weighted individual whole, half or third portions were crushed and thoroughly mixed for 2 minutes. For each aliquot, about 20 mg exactly measured of the fine powder were solubilised in a 20 ml volumetric flask with the appropriate solution. For INH-PZA, the solution consisted in the USP phosphate buffer pH 6.8. For RIF, methanol was spiked with ascorbic acid (0.2 mg/mL) and the volumetric flasks were covered by aluminium foil to protect the drug from the light [13]. To ensure the complete dissolution of the powder, test solutions were placed for 10 minutes in a thermostatic ultrasonic bath and immediately diluted 10 times to set the final concentration of INH, PZA and RIF at 7.5, 40 and 15 µg/mL respectively (corresponding to 100% of the expected labelled content for each drug). Finally, each solution was filtrated through a 0.45 µm Nylon membrane prior injection into the equilibrated HPLC system. Each measured concentration was corrected according to the exact amount of powder diluted into the volumetric flasks.

### Assessed parameters

To assess the uniformity of dosage units in a film-coated FDC tablet, containing more than 25 mg of each active ingredients but representing less than 25% of the whole tablet weight (apart from PZA), the USP recommends to estimate the statistical parameter “Acceptance Value” (AV) by the Content Uniformity (CU) method [11]. Several parameters must be measured and implemented to calculate this AV value, including the reference values, mean measured contents and standard deviations. The test needs to be performed on the first 10-units batch. If these failed, then not fewer than 20 extra units need to be further examined. Finally in the absence of any particular guidelines to assess the content uniformity of split portions, the test was translated and applied on half and third portions.

The content variation was expressed for each portion as the ratio between the measured value compared and the expected value, the later accounted by two different ways in this study. For the labelled variation, the measured content in each portion was compared with the labelled content. The labelled content in split portions was defined as being 50% of the whole tablet's content for a half tablet and 33.3% of whole tablet content for a third tablet. While in the weight-adjusted variation, individual split portion expected content was corrected by its weight proportion over the parent whole tablet weight. For example, a third tablet whose weight represented 43% of the parent whole tablet had an expected drug content of 43% (instead of 33%).

### Statistics

Statistical analysis for assessing the weight and content uniformity was performed using normality tests run on all the data sets (KS normality test and D'Agostino & Pearson Omnibus Normality Test). A Paired Student's t test was used to compare the means of weight of two halves from the same whole tablet. Analysis of variance (ANOVA) was used to compare the means of weight distribution between the third tablets from the same whole tablet. Standard unpaired t-test (Gaussian distribution and equal variance) was used to compare labelled content with weight adjusted content in halves and thirds portions. All statistical evaluations other than the AV calculation were performed on Prism 6 for Windows, version 6.01.

## Results

### Analytical method

Several monographs were evaluated to initially measure the content of INH, PZA and RIF, from either separate or FDC formulation. However, the recommended extraction buffer solution (phosphate buffer pH 6.8) provided a very poor extraction yield for RIF with the Vietnamese formulation. Therefore we adapted, for this study, an internally validated method for the detection of INH, PZA and RIF but developed for plasma and CSF. When the pH 6.8 phosphate buffer was kept for extracting INH-PZA, it was replaced by methanol for RIF providing a higher extraction yield. Secondly, in order to avoid using a long gradient to elute both polar and non-polar TB drugs (such as described in the USP), the analysis was split in two stages according to polarities, with a first aliquot analysed to measure INH and PZA concentrations, and a second aliquot to measure RIF concentration. Finally, a modification of the pH of the phosphate buffer mobile phase from 6.8 (USP monograph) to 4.2 enhanced the quality of the separation and reduced the run time.

### Weight uniformity

The mean measured weight (+/−SD) of whole tablet was 949.6 mg (+/−10.3). With respectively 1.08% and 5.43%, both the whole and half tablets passed the weight variation standard limit of 6.00% (expressed as the coefficient of variation or CV%). But the third fractions failed, rising by as much as twice the tolerated variation with 12.44%. While no significant difference was found between the weights of two halves from the same whole tablet (*p*-value = 0.251), a significant weight heterogeneity (*p* = 0.014) was shown for the third tablets (Table 1). In addition, the weight loss after splitting was significantly increased (*p*-value <0.0001) with 9.7 mg (±3.8 mg) for the third portions than it was for the halves with 2.0 mg (±1.7 mg).

**Table 1:** Weight of whole and split tablets expressed as percentage (%) of the measured mean of the whole tablets.

	Whole tablet (%)	Half tablet (%)	Third tablet (%)
Mean	100.00	50.22	32.92
Min	98.51	45.82	26.08
Max	101.91	55.12	43.97
CV%	1.08%	5.46%	12.44%
P-value		0.251^a^	0.014^b^

CV%: coefficient of variation (standard deviation/mean) x100, a: paired Student's t test, b: analysis of variance (ANOVA).

### Content uniformity and AV test

For the whole tablets, the content uniformity test for the three drugs passed after the first batch of 10 units. In halves and thirds portions, the test failed after 10 units, and failed again for the next 20 following units (Table 2). Corrected by weight adjustment, the values were found smaller, but the content uniformity failed for all drugs excepted for PZA in halves.

**Table 2:** Acceptance Values (AV) for the content uniformity according to the USP monograph 905 (for the three drugs in whole, half and third portions, the accepted limit was set up <15).

		Whole		Halves			Thirds		
	Drug	10 units		10 first units	20 next units		10 first units	20 next units	
Labelled content	INH	12.6	**Pass**	24.7	15.5	Fail	52.9	41.8	Fail
	PZA	8.63	**Pass**	21.1	15.2	Fail	45.5	30.3	Fail
	RIF	14.0	**Pass**	39.0	30.0	Fail	40.0	39.9	Fail
Weight adjusted content	INH			19.5	19.6	Fail	20.7	30.5	Fail
	PZA			18.8	12.9	**Pass**	22.1	17.7	Fail
	RIF			36.0	27.2	Fail	30.6	31.5	Fail

### Labelled variation content

The results of the content uniformity in whole tablets, using labelled content as the expected targeted quantities of INH, PZA and RIF showed mean values within the 85–115% USP proxy for the 10 whole tablets analysed. Furthermore, the variability of the drugs content remained low with a CV% of 5.75%, 3.08% and 4.58% for INH, PZA and RIF, respectively. In half-tablets, the content variations exceeded the tolerated variation range with a number of half-tablets outside of the range going from 3.3% for INH up to 46.7% for RIF (Table 3). In third-tablets, content variations were even greater, with a significantly increased number of units outside of the range, showing at least more than 33.3% of split tablets outside the USP proxy for the three drugs (Table 3).

**Table 3:** Summary of whole, halves and thirds tablets content variation expressed as the percentage of measured over the labelled quantities.

	Drug	Mean variation %	%CV	Variation range (%)	Number outside USP proxy
**Whole (n = 10)**	INH	101	5.19	97.6–105	0
	PZA	95.4	2.43	93.8–97.1	0
	RIF	93.4	3.94	90.7–96.0	0
**Halves (n = 30)**	INH	105	9.10	88.1––123	5 (16.7%)
	PZA	98.6	7.72	88.3–118	1 (3.33%)
	RIF	87.1	10.7	73.4–109	14 (46.7%)
**Third (n = 36)**	INH	107	17.1	67.8–137	18 (50.0%)
	PZA	101	15.0	72.6–138	12 (33.3%)
	RIF	88.0	16.7	54.3–112	13 (36.1%)

*USP proxy defined as 85*–*115% of the expected content*.

**Figure 1:**
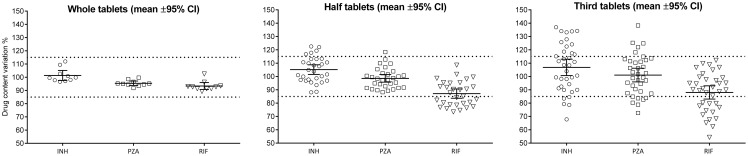
Labelled content variation in whole, half and third tablets. Results are presented as individual values and with mean and the 95% confidence interval of the mean for each drug and dataset. The dashed lines represent the 85–115% USP proxy.

The overall observation was a spread of the measured values around the expected means as soon as the splitting occurred (figure 1). As an example, for RIF, splitting the tablets moved the spread values towards a generalised under-dosing with a mean content (95% CI) of 87.13% (83.66%–90.60%) in the halves and 88.00% (83.03%–92.98%) in the third fractions.

### Weight-adjusted variation content

This adjustment decreased the number of split portions outside of the range (Table 4). But although the number of failed halves was reduced for INH (3/30, 10.0%), it still remained critical for RIF (12/30, 40.0%). A similar trend was found for the third portions, where the number of failed fractions decreased. However, the number of rejected fractions was still significant, particularly for RIF (11/36, 30.6%). The finding with RIF was that all rejected split units (halves and thirds) were also found below the lower RIF limit of 85%.

**Table 4:** Summary of halves and thirds tablets content variation using the weight-adjustment correction.

	Drug	Mean variation %	%CV	Drug content variation range (%)	Number outside USP proxy
**Halves (n = 30)**	INH	105	7.46	90.2–118	3 (10.0%)
	PZA	98.9	6.55	89.5–111	0
	RIF	87.3	9.16	73.7–102	12 (40.0%)
**Third (n = 36)**	INH	108	11.2	79.3–140	9 (25.0%)
	PZA	102	8.35	80.0–119	3 (8.332%)
	RIF	89.0	12.4	60.7–111	11 (30.6%)

*USP proxy defined as 85*–*115% of the expected content*.

## Discussion

### Weight variation

The tested whole tablets presented a negligible weight variation. In half portions, the small weight variability was attributed to the score line put on the whole tablets, which allowed dividing the tablet into two equal halves. Regarding the third units, the inability to produce identical fractions might be explained by the oval shape of the tablet, unscored for the third portions, thus with an increased risk to produce small fragments.

### Labelled content

Despite the high degree of quality of the FDC whole tablets, the drug content uniformity in the split portions for the three anti-TB drugs led to clinically relevant and unacceptable variation.

In halves, while PZA almost passed the USP content uniformity test (only one portion i.e. 3.3% was rejected), both INH and RIF fell outside of the USP expectations. Indeed the content variation for INH showed an over-dosing trend with all the rejected halves (n = 5, 16.7%) outside the upper-limit proxy, whereas for RIF, the opposite trend was found with an under-dosing drift shown by all the half portions (n = 14, 46.7%) outside the lower-limit proxy of 85%. Similarly in the third portions, poor homogeneity in the content uniformity was seen with a higher magnitude. While the variation of INH and PZA was extended at the lower and upper limits, all the rejected split third portions for RIF (n = 13, 36.1%) were found below the lower-limit of 85% variation. One extreme case was found with a third fraction, which showed only 54.3% of its expected content of RIF.

In halves, the score line allowed an identical split, but the drug content uniformity still varied in the split portions. A poor uniformity was observed when the tablets were split three ways for all drugs. However this could have been enhanced by the unequal splitting of the third portions from the parent tablet. To evaluate the impact of the asymmetrical distribution of the three drugs within parent portions, an analysis of the correlation between the measured drug content and the split portion's weight was performed (described in details in Tables S1). In halves, significant correlations were found for all drugs between the weight of the portion from the parent tablet and its measured content (*p*-values <0.01 for INH, PZA and RIF). The correlation coefficients were consistent for the three drugs with a Pearson's r value (±95%CI) of 0.57 (0.26–0.77), 0.56 (0.24–0.76) and 0.47 (0.14–0.71) for INH, PZA and RIF, respectively. In the thirds, the correlation was more important with all *p*-values <0.0001 and the Pearson's r values (±95%CI) of 0.79 (0.63–0.89), 0.85 (0.73–0.92) and 0.69 (0.47–0.83) for INH, PZA and RIF, respectively. Both split portions showed a significant correlation, meaning a consistent repartition of the three drugs within the tablets. The implications of these values are difficult to interpret, especially the difference between tablets split into halves and thirds. Hill and coll. in a study arbitrarily set a Pearson's coefficient below 0.7 as a non-uniformity limit value [14]. However in our study this would mean that the tablets split into thirds showed content uniformity but not the halves, which clearly does not make clinical sense. This may be explained by the analytical method: in our study, a sample of 20 mg from the crushed and mixed split portions was analysed. Those 20 mg represented a higher proportion of the split thirds (mean = 6.48%) than it did for the halves (mean = 4.21%). Consequently, the spread of the measured contents around a regression model was likely to be more accurate and representative when the weight ratio of the sample over the whole portion was increased. This correlation could only be used to compare the uniformity of the three drugs within the same portions, or would be relevant to compare, by the same method, the drug content from different formulations.

### Weight adjusted content

**Figure 2:**
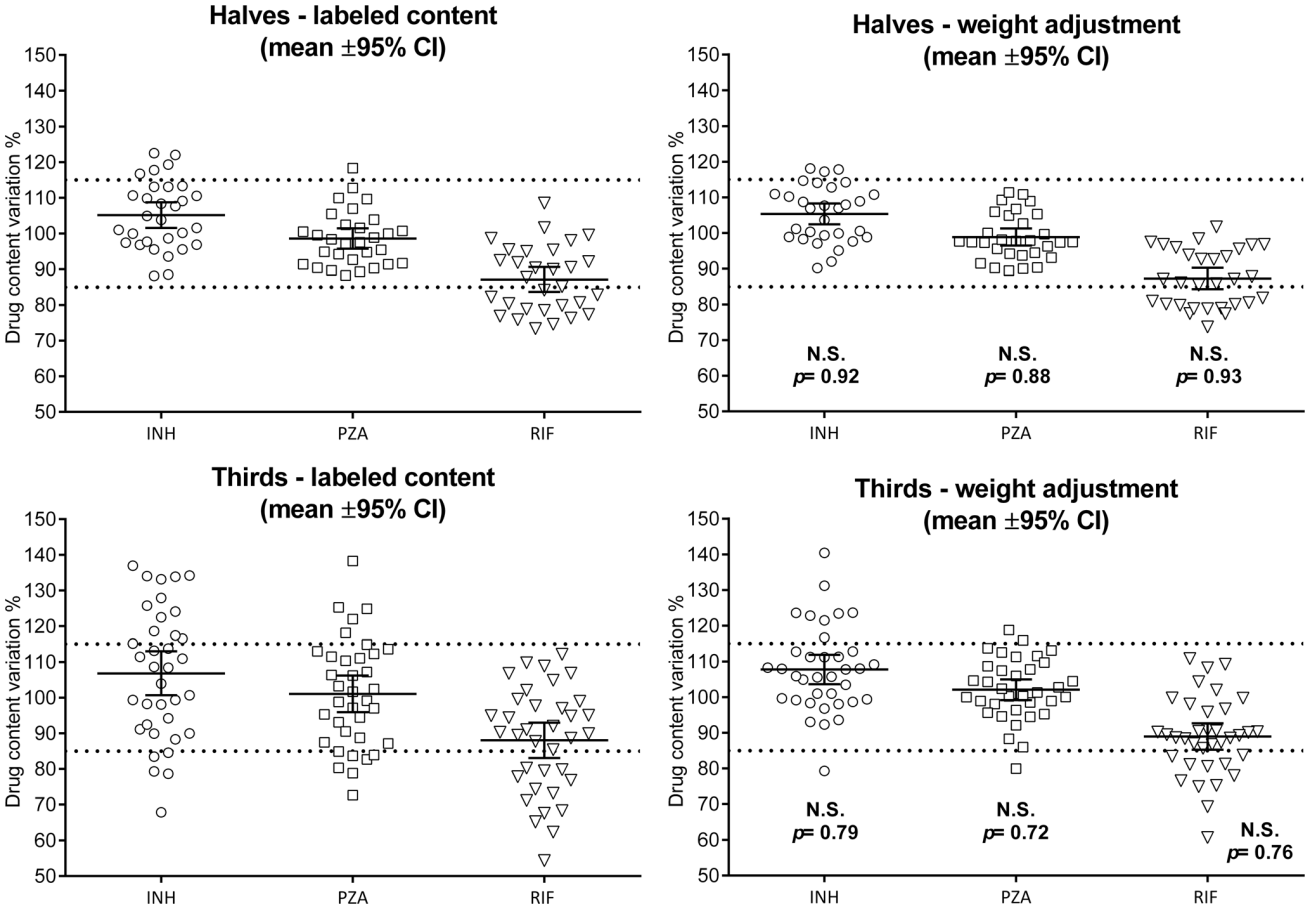
Labelled content versus weight-adjusted content in halves and third tablets. Results are presented as individual values and with mean and 95% confidence interval of the mean for each drug and dataset. The dashed lines represent the 85–115% USP proxy. P-values were calculated with a standard unpaired t-test comparing labelled content with the weight adjusted content - NS stands for “Not Significant”.

In order to correct the inaccuracy of splitting the tablets in equal parts, the expected content was adjusted by the weight ratio of the portion over the parent whole tablet. As seen on figure 2, the effect of the weight adjustment strategy remained not significant for the 3 drugs in halves and thirds portions. However, as seen with the CV% values for halves and thirds (Table 4), the content variation expressed with the weight-adjustment showed a decreased spread of the values. This effect was maximised for the third tablets. The range of content variation was found narrower, due to the reduced variation of the outliers for which the labelled expected content was biased by the irregularity of their weight.

Nevertheless, the conclusion remained the same: except for PZA when split in two, the content uniformity tests failed for all drugs in half and third portions. A similar trend was found, with an over-dosing for INH, and an under-dosing for RIF. In a clinical context, the adjustment of the content according to the weight may allow a better control of dose delivery. However this is programmatically impossible to implement as it would require health care staff to weigh a whole tablet, then weigh the split portion and finally correct the expected content by the weight ratio. This would be impossible in the real world settings of TB delivery progammes globally. Moreover, it would waste the unused portion.

Although the implementation of FDC has contributed to what success there has been in the control of TB globally, further work is required to offer the best treatment in childhood TB. As shown by our study, the splitting process significantly affects the quality of the pharmaceutical formulation and only a paediatric formulation would overcome this problem. Oral solutions can be seen as advantageous for the treatment delivery in the youngest patients because of the body weight scalability using a syringe and markings. However, oral forms are also more expensive to produce [15], present storage issues and are currently not available in fixed-dose combinations for TB. On another hand, dispersible or chewable tablets present several advantages over the liquid forms. The pharmaceutical production of FDC tablets for adults seems globally well controlled and only few adjustment are required to produce tablets for smaller body weight bands (i.e. 5 kg) with a child-friendly taste (if a dispersible form is considered). In some cases, it was shown that a vast majority of caregivers and children preferred the tablets over the liquid form, mostly because of swallowing and vomiting problems [16]. The advantage of a new paediatric formulation in TB would need to be assessed in a formal pharmacokinetic analysis of the anti-TB drugs in individuals treated with split tablets versus a dispersible form. A formal compliance assessment of both children and care givers would also provide extremely useful information for a better design of new formulations.

## Conclusion

To our knowledge, this is the first investigation of the content uniformity in split fractions of a fixed-dose combination containing INH, PZA and RIF. Our study showed that the content uniformity of INH, PZA and RIF in the FDC is disrupted as soon as the whole tablet is broken. Even if the tablets were scored for halves, the degree of variability was still unacceptable for the three drugs and this was more marked when the tablets were split three ways. This would led to children received an inaccurate dose regimen. This has major implications for a treatment effect but also by potential under dosing encouraging the selection of drug resistant mutants of Mycobacterium TB. This study highlighted the clinically relevant problem of an inappropriate oral therapy using split adult FDC tablets in the treatment of childhood TB, and the need to develop an affordable child-friendly formulation of FDC for the first-line anti-TB drugs.

## Supporting Information

Tables S1 Supplementary material. Raw values of weighting, measured concentrations (HPLC) and extrapolated content and content variations in whole, half and third tablets. The full statistical comparison of labelled versus weighted contents is presented for INH, PZA and RIF. The correlation and linear regression test between portion's weight and content are also presented.
(XLSX)
